# Key Factors in Fluid Irrigation Control: A Comparative Study of Arthroscope and Monoportal Scope in Biportal Spine Surgery

**DOI:** 10.7759/cureus.72738

**Published:** 2024-10-30

**Authors:** Takeshi Kaneko, Ryoji Tominaga, Hiroki Iwai, Yuichi Takano

**Affiliations:** 1 Spine Surgery, Inanami Spine and Joint Hospital, Tokyo, JPN; 2 Spine Surgery, Iwai Orthopaedic Hospital, Tokyo, JPN

**Keywords:** assisted full endoscopic spine decompression, lumbar spinal canal stenosis, lumbar spinal decompression, monoportal spine surgery, unilateral biportal endoscopic surgery

## Abstract

The purpose of this retrospective study was to compare patient satisfaction and irrigation fluid usage between arthroscopy-based Biportal Endoscopic Decompression (A-BED) and monoportal scope-based biportal decompression, also known as Assisted Full-Endoscopic Spine Surgery (AFESS). A total of 89 patients (52 A-BED, 37 AFESS) who underwent either procedure between September 2020 and April 2024 were included in the study. While arthroscopic scopes have traditionally been used in biportal surgeries, the monoportal scope offers the advantage of self-contained fluid management, allowing for more efficient irrigation. Fluid usage was measured by the number of 2 L saline bottles used per hour of surgery, and postoperative pain was assessed using a Numeric Rating Scale (NRS). Patient satisfaction at two weeks was similar between the groups (p < 0.71), while the AFESS group demonstrated significantly lower irrigation fluid usage (p < 0.001). Additionally, AFESS showed shorter operative times (84.8 min vs 99.3 min, p < 0.01). The monoportal scope's efficient fluid evacuation reduced the need for excess irrigation, contributing to reduced medical resource consumption without compromising patient satisfaction or safety. There was no significant influence of patient characteristics such as age, gender, or BMI on fluid usage, suggesting that the difference is primarily attributed to the surgical technique. No significant differences in complication rates or reoperations were observed between the two groups. AFESS thus shows potential for reducing irrigation-related risks and improving surgical efficiency while maintaining clinical outcomes comparable to the traditional method.

## Introduction

Unilateral Biportal Endoscopic (UBE) surgery has emerged as a promising method for spinal decompression procedures [1-4]. UBE involves the use of two small portals: one for the endoscope and another for surgical instruments, allowing for improved visualization and reduced muscle dissection compared to traditional open surgery [5-6]. The effectiveness of UBE in decompression surgeries has been well-documented, with studies showing improved outcomes due to better visualization and minimal tissue damage [7]. Compared to traditional open surgery, UBE provides a wider field of view, enabling surgeons to more precisely target pathological structures. Traditionally, biportal endoscopic spine surgery has utilized arthroscopic scopes, which contain a narrow tube housing a light source and camera [8]. However, these scopes lack an instrument insertion channel and are limited to inflow irrigation only. This limitation often necessitates increased irrigation fluid usage and can lead to potential complications related to fluid management [9,10]. Recently, a novel approach has been developed: the adaptation of monoportal scopes to biportal procedures, resulting in Assisted Full-Endoscopic Spine Surgery (AFESS) [11]. This technique aims to combine the benefits of biportal surgery with improved fluid management and visualization capabilities. The self-contained irrigation system in the monoportal scope allows for both inflow and outflow, potentially reducing irrigation fluid use and related complications. Despite the theoretical advantages of AFESS, there is a lack of direct comparisons between this novel technique and the traditional arthroscopy-based Biportal Endoscopic Decompression (A-BED). Such a comparison is crucial to understand the relative merits of each approach in terms of surgical efficiency, patient outcomes, and resource utilization. Therefore, the primary aim of this study was to directly compare A-BED and AFESS, with a particular focus on irrigation fluid usage, operative time, and patient satisfaction. We hypothesized that AFESS would demonstrate reduced irrigation fluid usage without compromising surgical outcomes or patient satisfaction. Additionally, we sought to investigate whether patient characteristics such as age, gender, or BMI influenced the effectiveness of either technique.

## Materials and methods

Data source and study population

We retrospectively analyzed 89 patients who underwent A-BED or AFESS between September 2020 and April 2024 at Inanami Spine and Joint Hospital, Tokyo, Japan. The sample size was determined using power analysis to achieve an 80% power with an alpha level of 0.05, ensuring the ability to detect a significant difference in fluid usage between the two groups. This sample size, while slightly below our initial target of 90, proved sufficient for detecting significant differences between the two techniques. The inclusion criteria were adults (≥18 years) with lumbar spinal canal stenosis presenting with cauda equina syndrome or radicular symptoms who had failed conservative treatment for at least six weeks. We excluded patients with multi-level decompression needs, trauma-related pathologies, and previous lumbar surgery at the same level. The primary focus was to compare irrigation fluid usage, recorded as the number of 2 L saline bottles used per hour of surgery. Postoperative pain was assessed using the Numeric Rating Scale (NRS) the day after surgery, and patient satisfaction was measured on a 0 to 10 scale, with higher scores indicating greater satisfaction. These measurements are part of the standard A-BED/AFESS treatment protocol to consistently evaluate postoperative outcomes and patient satisfaction across all cases. This standardized approach ensures uniform assessment and facilitates comparative analysis between the two techniques. We evaluated perioperative complications, including water-related symptoms such as neck pain and seizures, as well as postoperative complaints and changes in preoperative and postoperative C-reactive protein (CRP) levels. Fluid usage was analyzed with logistic regression to assess its relationship with procedure type, age, gender, and BMI. The choice between AFESS and A-BED was based on surgeon preference and equipment availability, with efforts made to balance group sizes.

Statistical analysis

Patient satisfaction and fluid usage rates were compared using chi-square tests for categorical variables and Wilcoxon/Kruskal-Wallis rank tests for continuous variables. A multivariable logistic regression model, adjusting for age, gender, and BMI, was used to assess changes in fluid usage. All analyses were conducted using Stata version 16 (StataCorp, College Station, TX), with p-values < 0.05 considered statistically significant.

Surgical procedure

The surgical procedure for both A-BED and AFESS was performed as previously described by Kaneko et al. [11] under general anesthesia with the patient in the prone position. The 2 L saline solution bottle was placed about 50 cm above the surgical field. The procedure begins by identifying the inner edge of the superior articular process on the preoperative CT axial view, drawing a guiding line parallel to the spinous process to prevent disorientation. The cranial camera portal is created using fluoroscopic guidance and is positioned near the lower edge of the cranial vertebral body, while the walking portal is placed 2-2.5 cm caudal. Initially, the monoportal scope (VERTEBRIS, Richard Wolf GmbH, Knittlingen, Germany) or the A-BED arthroscopic scope (HOPKINS Telescope 30°, 4 mm, Karl Storz, Germany) identifies the lamina, and the pencil dilator is inserted through the walking portal to switch to biportal. The lower edge of the lamina is drilled to create a groove, and a chisel may be used if needed. The superior articular process is then exposed, and the ligamentum flavum is removed in one piece if possible. The main difference between A-BED and AFESS is the scope type: A-BED uses an arthroscopic scope, whereas AFESS uses a monoportal scope, which allows for the use of the working channel. In A-BED, tasks cannot be performed through the camera portal, but the surgical methods are otherwise similar.

## Results

A total of 89 patients were included in this study, with 52 in the A-BED group and 37 in the AFESS group. The most common surgical level in both groups was L4/5. Patient characteristics are shown in Table 1.

**Table 1:** Patients’ characteristics The Mann-Whitney U test was used for age, operative time, and BMI. Gender was compared across the types of scopes using the chi-square test.

	Total	Monoportal scope	Arthroscopic scope	P-value
Number	89	37	52	P<0.11
Gender (male)	53	23	30	P<0.83
age	67.2 (32-86)	64.9 (32-86)	68.8 (40-86)	P< 0.11
BMI	24.2 (15.1-35.9)	23.7 (15.1-35.9)	23.2 (17.5-35.3)	P<0.93
Operating time (min)	93.3 (43-145)	84.8 (43-145)	99.3 (56-134)	P<0.05
Location (No)				
L2/3	9	1	8	
L3/4	10	2	8	
L4/5	66	32	34	
L5/S	4	2	2	

The mean age was 68.8 years (40-86) in the A-BED group and 64.9 years (32-86) in the AFESS group, with 83% of patients being male. The mean BMI was 23.2 (17.5-35.3) for A-BED and 23.7 (15.1-35.9) for AFESS. The AFESS group demonstrated significantly shorter operative times, averaging 84.8 minutes (43-145), compared to the A-BED group's 99.3 minutes (56-134). Postoperative results showed no significant differences in patient satisfaction at two weeks (P < 0.71), CRP levels (P < 0.55), or wound pain (NRS) on postoperative day 1 (P < 0.90) between the A-BED and AFESS groups. However, the AFESS group demonstrated significantly lower irrigation fluid usage per hour compared to the A-BED group (P < 0.00). No water-related symptoms and dural tears were observed in either group, and there was one case of re-operation due to postoperative hematoma in each group (Table 2).

**Table 2:** Comparison of surgical outcomes and complications between AFESS and A-BED techniques AFESS: Assisted full-endoscopic spine surgery; A-BED: Arthroscopy-based biportal endoscopic decompression; CRP: C-reactive protein.

	Monoportal scope	Arthroscopic scope	P-value
Water consumption (L/ hour)	2.43 (1.9-3.6)	2.89 (1.3-4.8)	P< 0.001
CRP rate (post/pre)	4.24 (0-33.4)	4.49 (0.18-27.1)	P< 0.55
One day postoperative NRS	4.49 (0-9)	4.56 (0-10)	P< 0.90
Two week patient satisfaction	7.50 (2-10)	7.10 (0-10)	P< 0.71
Complications			
Water-related complain	0	0	
Dural tear	0	0	
Hematoma	1	1	

Adjusted odds ratios are shown in Table 3. The type of surgical procedure was the most significant factor affecting irrigation fluid usage (odds ratio (OR) 9.52, 95% confidence interval (CI) 3.32, 27.35), with the AFESS technique leading to lower fluid consumption. Other factors, including age ≥ 67.2 years (OR 2.66, 95% CI 0.91, 7.83), male patients (OR 0.52, 95% CI 0.17, 1.56), and BMI ≥ 24.2 (OR 2.14, 95% CI 0.74, 6.19), were not statistically significant in relation to irrigation fluid usage.

**Table 3:** Logistic regression analysis of factors influencing irrigation fluid usage The dependent variable is high irrigation fluid usage. Odds ratio (OR) > 1 indicates higher odds of high fluid usage. Age and BMI were dichotomized using the overall mean values of the study population (67.2 years for age and 24.3 for BMI).

Variable	Odds ratio (OR)	95% Confidence Interval	P-value
Method (Arthroscopic based)	9.52	(3.32, 27.35)	P< .0
Age_Mean (> 67.2)	2.66	(0.91, 7.83)	P< 0.075
Sex (male)	0.52	(0.17, 1.56)	P< 0.242
BMI_Binary ( >24.3)	2.14	(0.74, 6.19)	P< 0.161

## Discussion

Our findings demonstrate that AFESS achieves comparable patient satisfaction to A-BED while significantly reducing irrigation fluid usage, as evidenced by a lower average number of saline bottles used per hour of surgery in the AFESS group (P < 0.001). Since 1996, arthroscopic scopes have been widely used for biportal spine surgery [12]. A significant challenge has been inadequate fluid evacuation from the working portal, often necessitating portal enlargement and increased irrigation volume, leading to higher fluid usage. In contrast, the monoportal scope used in AFESS allows for more efficient fluid evacuation, reducing the need for excess irrigation while maintaining similar surgical outcomes. Proper irrigation management is crucial in endoscopic spine procedures due to potential risks associated with fluid extravasation. Fluid extravasation during arthroscopic procedures, while potentially life-threatening, is usually managed conservatively [9]. Vargas et al. highlighted specific risks such as headaches, neck pain, and seizures due to irrigation fluid entering the spinal canal, emphasizing the importance of proper management of irrigation pressure, flow, and volume [10].

In our results, no significant correlation was found between age, gender, BMI, and irrigation fluid usage, suggesting that the surgical technique itself is the most influential factor. However, it's important to note that our dataset did not include patients with BMI exceeding 30. While our findings indicate no significant relationship between BMI and fluid usage for non-obese and mildly obese patients, we cannot extrapolate these results to severely obese individuals. Future studies should specifically evaluate patients with BMI > 30 to comprehensively assess whether severe obesity affects irrigation fluid dynamics in both AFESS and A-BED procedures.

Water dynamics in endoscopic spine surgery may be influenced by factors such as cannulation length and BMI [13]. A study measured irrigation fluid pressure during spinal surgery and identified that these factors significantly influenced water pressure, with longer cannulation and higher BMI leading to increased pressure. Maintaining water pressure at safe levels is critical to preventing complications related to fluid dynamics, including the potential risk of fluid entering unintended body compartments due to insufficient evacuation from the working portal or increased hydrostatic pressure. No water-related complications or dural tears were observed in either group in this study. However, if a dural tear had occurred, water-related complications could not be ruled out. In similar procedures, dural tears can lead to cerebrospinal fluid leakage, which may require surgical repair or conservative management depending on the severity. Studies have reported that timely identification and appropriate intervention are crucial to minimize the impact of such complications. In previous cases with A-BED, there have been reports of rare complications such as abdominal pain due to retroperitoneal fluid retention [14].

Our study demonstrates that AFESS offers several key advantages over traditional biportal techniques. AFESS achieves comparable clinical outcomes, with similar patient satisfaction and surgical effectiveness as traditional methods. However, its significant advantage lies in the substantial reduction of irrigation fluid usage, enhancing patient safety and contributing to resource conservation. The improved fluid management is attributed to the monoportal scope used in AFESS, which allows for more efficient fluid evacuation, potentially reducing complications related to fluid extravasation. We observed shorter operative times with AFESS. While this could be partially attributed to increased familiarity with the technique following initial experience with A-BED, it nonetheless suggests improved efficiency in the surgical process. It's important to note that despite these advantages, the core surgical procedure remained largely unchanged, with the primary difference being the type of scope used. This minimizes the learning curve for surgeons transitioning to AFESS and ensures that the benefits, particularly in terms of improved water irrigation management, are achieved without significant alterations to the overall surgical technique. However, several limitations of this study should be acknowledged. The retrospective nature of the study may introduce selection bias, and the lack of long-term follow-up data limits our ability to assess long-term outcomes and complications. Additionally, the measurement of saline usage in 2 L units lacks precision, and future studies should consider more accurate methods of fluid measurement.

## Conclusions

AFESS demonstrates significant advantages in terms of reduced irrigation fluid usage compared to A-BED, while maintaining comparable patient satisfaction and potentially improving surgical efficiency. These findings suggest that AFESS may represent a safer and more efficient approach to minimally invasive spine surgery. However, further research, particularly prospective, multi-center studies with long-term follow-up, is needed to fully establish the role of AFESS in the evolving field of endoscopic spine surgery.
